# The equity dimension in evaluations of the quality and outcomes framework: A systematic review

**DOI:** 10.1186/1472-6963-11-209

**Published:** 2011-08-31

**Authors:** Pauline Boeckxstaens, Delphine De Smedt, Jan De Maeseneer, Lieven Annemans, Sara Willems

**Affiliations:** 1 Department of Family Medicine, Ghent University, UZ-1K3 De Pintelaan 185, 9000 Ghent, Belgium; 2 Center for Health Economics, Department of Public Health, Ghent University, UZ-1K3 De Pintelaan 185, 9000 Ghent, Belgium

## Abstract

**Background:**

Pay-for-performance systems raise concerns regarding inequity in health care because providers might select patients for whom targets can easily be reached. This paper aims to describe the evolution of pre-existing (in)equity in health care in the period after the introduction of the Quality and Outcomes Framework (QOF) in the UK and to describe (in)equities in exception reporting. In this evaluation, a theory-based framework conceptualising equity in terms of equal access, equal treatment and equal treatment outcomes for people in equal need is used to guide the work.

**Methods:**

A systematic MEDLINE and Econlit search identified 317 studies. Of these, 290 were excluded because they were not related to the evaluation of QOF, they lacked an equity dimension in the evaluation, their qualitative research focused on experiences or on the nature of the consultation, or unsuitable methodology was used to pronounce upon equity after the introduction of QOF.

**Results:**

None of the publications (n = 27) assessed equity in access to health care. Concerning equity in treatment and (intermediate) treatment outcomes, overall quality scores generally improved. For the majority of the observed indicators, all citizens benefit from this improvement, yet the extent to which different patient groups benefit tends to vary and to be highly dependent on the type and complexity of the indicator(s) under study, the observed patient group(s) and the characteristics of the study. In general, the introduction of QOF was favourable for the aged and for males. Total QOF scores did not seem to vary according to ethnicity. For deprivation, small but significant residual differences were observed after the introduction of QOF favouring less deprived groups. These differences are mainly due to differences at the practice level. The variance in exception reporting according to gender and socio-economic position is low.

**Conclusions:**

Although QOF seems not to be socially selective at first glance, this does not mean QOF does not contribute to the inverse care law. Introducing different targets for specific patient groups and including appropriate, non-disease specific and patient-centred indicators that grasp the complexity of primary care might refine the equity dimension of the evaluation of QOF. Also, information on the actual uptake of care, information at the patient level and monitoring of individuals' health care utilisation tracks could make large contributions to an in-depth evaluation. Finally, evaluating pay-for-quality initiatives in a broader health systems impact assessment strategy with equity as a full assessment criterion is of utmost importance.

## Background

**Figure 1:**
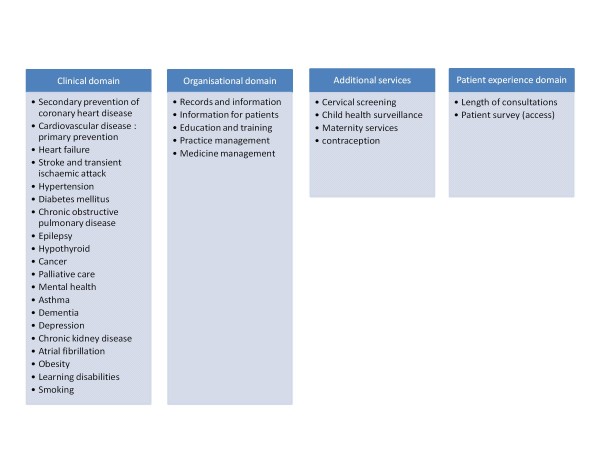
**Domains of the QOF (2009) **[64].

The implementation of pay for performance systems (P4P) for primary care is rising internationally. The Quality and Outcomes Framework (QOF) is unarguably the most comprehensive national primary care pay for performance scheme in the world. It was introduced in April 2004 as a system for the evaluation, management and payment of general practitioners (GPs) in the National Health Service (NHS) in England, Wales, and Scotland and was part of the new general medical services (GMS) contract. QOF replaced various other fee arrangements and ties up to 25% of the income of primary care practices to the quality of delivered care. In the original 2004 contract, GPs could accumulate up to 1050 QOF points depending on their level of achievement with respect to 146 indicators. The criteria are grouped into four domains: clinical, organisation of care, patient experiences and additional services (Figure 1). At the end of the financial year, the total number of points achieved by the practices is collected by the QMAS, which converts the total points into a payment amount for the practice. After its introduction in 2004, QOF underwent several adaptations in the number and nature of indicators and in the relative impact of the four domains. For 2009/10, the clinical indicators represented 70% of the achievable points, and practices were paid on average 126,77 pounds for each point achieved.

Changes in the policy or the organisation of health care require that the effects on all dimensions of care be monitored [1]. Therefore, an explicit assessment of equity in health care and moreover, of equity in health [2-4], is of utmost importance.

**Figure 2:**
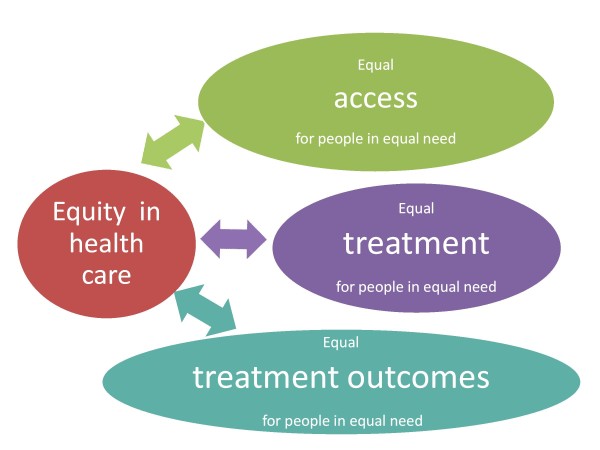
**Conceptual framework for equity in health care**.

Measuring equity in health care is a true challenge, not least because there is no consensus on how to define and measure the concept. In the literature regarding the conceptualisation of equity, some common ground can be found by the recognition of three domains: 1°, equal access to care for people in equal need 2°, equal treatment for people in equal need and 3°, equal treatment outcomes for people in equal need (Figure 2). Despite its simplification of the nature of equity, the definition that these three domains provides is a useful framework that delineates where inequities in health care may arise [5].

Regarding access to health care, the definition of the concept is highly contingent on the context within which the analysis takes place. Goddard and Smith define access to health care as "the ability to secure a specified range of services at a specified level of quality, subject to a specified maximum level of personal inconvenience and costs, whilst in the possession of a specified level of information" [6]. Concerning the range of services provided, the aspect "availability of equal services for people in equal need" is widespread in the literature on equity [6,7]. This aspect refers to the fact that factors such as age, sex and income should not dictate that people with similar needs enter different doors (e.g., public versus private providers) or be treated differently in terms of the type or intensity of services provided [7]. Quality of service is also an intrinsic element of access because poor quality in terms of the structure, the care provided or the outcome might compromise access [6]. Concerning the aspects of personal inconvenience, cost and information, there might be considerable variation in the personal costs of using services (such as user fees and transportation costs) and in the awareness of the availability and efficacy of services (e.g., because of language or cultural differences). Although completely equalising these aspects is not feasible, there must be some point where differences in costs and information distribution become unacceptable [6,8]. In assessments of equity in treatment and treatment outcomes, the interaction between patient and provider plays a major role. Variations that arise from this interaction depend on the knowledge, skills, preferences, perceptions, attitudes and prejudices of both patient and health care provider [6]. Moreover, the wider social determinants of health such as the social circumstances in which people live and work might contribute to inequity in treatment and treatment outcomes. For example, unequal recovery rates in different social groups may occur even when there is no inequity in their access or the treatment that has been provided [2]. For these reasons, analysing equity in treatment and treatment outcome is complex and not always feasible [2]

One of the central principles in the conceptualisation of equity is 'need' [2]. The 'taxonomy of need' identifies 4 domains [5,9]: 'normative need' (defined by an expert or professional according to his/her own standards), 'felt need' (where people identify what they want, which might be limited or inflated by people's awareness and knowledge about what could be available), 'expressed need' (namely, felt need that has been turned into an expressed request, which can therefore be conceptualised as demand for care and-if the demand is fulfilled-care utilisation) and 'comparative need', (defined by comparing the care use rates of different groups of people where the group who uses the least is defined as being in need). This last approach simply compares and makes no judgments about the appropriateness or the adequacy of the use in the group with the highest rates [5,9].

This paper aims to describe the evolution of pre-existing (in)equity in health care in the period after the introduction of QOF. One of the unintended consequences of QOF might be that providers are encouraged to select only healthy and uncomplicated patients for whom targets are easily reached. To counter this fact, exception reporting has been introduced in the contract. It allows GPs to exclude patients to whom a quality indicator does not apply (e.g., women with a mastectomy are excluded from the mammography achievement score count), or for whom other considerations take precedence [10]. As exception reporting might be (ab)used to exclude specific patient groups, we also aim to describe inequities in exception reporting.

## Methods

### Search strategy

On 01/11/2009, MEDLINE and Econlit were systematically searched to identify publications on the evaluation of QOF. The following search strings were used: ("Quality and outcomes framework" OR "Pay for performance" OR ("contract" AND "primary care")) AND ("UK" OR "England" OR "Wales" OR "Scotland"). The search was limited to publications from 1/1/2004 to the present. Because the Quality and Outcomes framework is part of the UK healthcare setting, we limited the search to publications in English.

This search resulted in 317 publications, which were then screened for relevance and methodological suitability to answer the research question. The reference lists of the publications that were finally included in this study were screened but did not identify additional publications.

### Selection procedure of the publications

**Figure 3:**
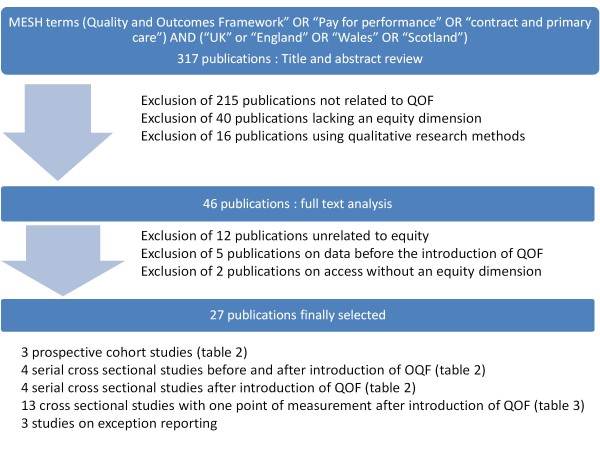
**Flowchart of the selection process**.

The titles and abstracts of the 317 identified studies were screened for their focus on the Quality and Outcomes Framework and for explicit references to equity related concepts (such as inequality, inequity, social differences, disparities, inverse care law) or to subgroups (social, ethnic, age or gender groups). A total of 215 publications were excluded because they were not related to the evaluation of the Quality and Outcomes Framework. Furthermore, 40 publications were excluded because they focused on (disease-) specific achievement scores without adopting any equity dimension in their evaluation. Additionally, 16 publications using qualitative research methods were excluded because they reported on the nature of the consultations or on the perspectives of patients or healthcare providers within QOF. The remaining 46 publications appeared to be related to equity in healthcare by explicit reference to equity related concepts or to subgroups in the title or abstract. Next in the selection process, an independent full text analysis of these 46 publications was performed by two researchers (SW and PB) to confirm the presence of the equity dimension. Publications labelled as "doubtful relevance concerning equity" by one of the two reviewers were discussed until a consensus was reached. Nineteen publications were rejected in this phase. Twelve publications appeared to be unrelated to equity because no subgroups had been defined [11-15], the subgroups were only related to a geographical area [16,17] or because of unsuitable methodology upon which to base conclusions regarding the defined subgroups [18-22]. Five publications appeared to be based on data collected before the introduction of QOF [23-27]. Two studies reporting on access to a GP within QOF were excluded after discussion because they did not evaluate any discrepancy between subgroups or address differences between users and non-users. Eventually, 27 studies were selected: 24 publications were labelled as relevant to assessing the equity dimension in the Quality and Outcomes Framework [28-51] and 3 focus on the effects of exception reporting on equity [10,52,53] (Figure 3).

### Data extraction

To assess the equity dimension in the publications evaluating QOF and to extract the data from the selected publications, a conceptual framework on measuring equity in health care was used (Table 1). This framework was developed by SW and PB based on the equity assessment literature (see also the Introduction). Data were extracted in duplicate by the same two reviewers. In cases of lack of clarity or differing findings between the two reviewers, the publications were discussed in detail by the team until clarity or consensus was reached.

**Table 1:** Data extraction template

General information	Equity aspects	Methods
Author	**Type of equity addressed**	**Study design**
Affiliation	- Access	- Cross sectional
Country	- Treatment	- Serial cross-sectional
Healthcare setting	- Outcomes	- Longitudinal
Journal	Conceptualisation of **need**	**Time frame **(pre/post contract)
Title	**Groups considered**	**Study population**
**Aim**	- Socio-economic status	**Datasource**
	- Ethnicity	- QOF database
	- Age	- Wandsworth Primary Care Based Registers
	- Gender	- Scottish Program for improving clinical effectiveness in primary care (SPICE) database
		**Outcome variable**
		Level of analysis
		- Patients
		- Practices
		- Primary care trusts

### Statistical analysis

This paper systematically summarises the statistically significant results as provided in the selected publications. Only statistically significant results are reported. A meta-analysis of the selected papers is not in the scope of this paper.

## Results

### General characteristics of the selected studies

#### Quality appraisal

To generate strong evidence concerning the impact of health care reforms on (in)equity in care, randomised controlled trials or studies over time with a concurrent control group would be required. However, none of the included studies applied this type of design. Because observational studies are the main source of information on QOF, we can only report on studies with rather weak evidence. However within the group of 24 papers selected to describe the evolution of (in)equity after the introduction of QOF, two subgroups can be identified with different levels of quality: the serial cross-sectional studies (Table 2) and the simple cross-sectional studies (Table 3). The serial cross-sectional studies (n = 11) have an appropriate design to describe the evolution of (in)equity in health care: three have measurements before and after the introduction of QOF linking individual data of a cohort over the years [28-30], 4 have measurements before and after the introduction of QOF [31-34] and 4 report on data solely collected after the introduction of QOF to illustrate the evolution of possible gaps in the years after the introduction of QOF [35-38]. Thirteen studies have a simple cross-sectional design with one point of measurement after the introduction of QOF [37,39-50]. They can illustrate the presence or absence of a gap but cannot describe its evolution.

**Table 2:** Serial cross-sectional studies with measurements both before and after introduction of QOF

Study	Study design	Condition/Indicator	Cross sectional analysis after introduction of the Quality and Outcomes Framework
***Deprivation***			

Millet 2007. Impact of a pay-for performance incentive on support for smoking cessation among people with diabetes	Longitudinal Repeated measurements Pre- and post contract 2003 and 2005 Effects of deprivation studied adjusting for age, sex, ethnic background and practice level clustering.	Diabetes recorded smoking status Smoking cessation advice smoking prevalence	Recorded smoking status: No gap documented post contract Recorded smoking status: No gap documented post contract Smoking: No gap documented post contract

Millet 2008c. Impact of a pay for performance on ethnic disparities in intermediate outcomes for diabetes: longitudinal study	Longitudinal repeated measurements Pre- and post contract 2000 and 2005-2006	Diabetes HbA1c & BP measured HbA1c & BP levels	Change in BP levels: indifferent for neigbourhood deprivation Change in HbA1c level: indifferent for neigbourhood deprivation

Mc Govern 2008b. The effect of the UK incentive based contract on the management of patients with coronary heart disease in primary care	Serial cross sectional Pre and post contract 2000 and 2005	CHD^1^ 11 indicators	Increasing gap from 1/11 to 4/11 CHD indicators pro less deprived.

McGovern 2008 Introduction of a new incentive and target based contract for family physicians in the UK: good for older patients with diabetes but less good for women	Serial cross sectional Pre and post contract 2000 and 2005	DM^2^ 8 indicators	Decreasing gap from 2/8 to 1/8 DM indicators pro less deprived

Simpson 2006. Effect of the UK incentive based contract on the management of patients with stroke in primary care	Serial cross sectional Pre and post contract 2004 and 2005	CVD^3^ 9 indicators	Increasing gap from 1/9 to 3/9 CVG indicators pro less deprived

Crawley 2009. Impact of pay for performance on quality of chronic disease management by social class group in England	Serial cross sectional Pre contract 2003 Post contract 2006	CHD - BP achievement - Use of antihypertensives - Cholesterol achievement - Use of lipid lowering drugs Diabetes - BP achievement - Use of antihypertensives - Cholesterol achievement - Use of lipid lowering drugs - Hba1c achievement - Use of oral hypoglycaemic agents Hypertension - BP achievement - Use of antihypertensives	Emerging gap pro non-manual occupations No gap documented No gap documented No gap documented No gap documented No gap documented No gap documented No gap documented Decreasing gap to a non significant difference pro non-manual occupations No gap documented No gap documented No gap documented

Ashworth 2008. Effect of social deprivation on blood pressure monitoring and control in England: a survey of data from the quality and outcomes framework	Serial cross sectional Post contract 2004-2005; 2005-2006 and 2006-2007	5 chronic conditions BP^4 ^monitoring BP target values	BP monitoring: gap narrowed to a negligible difference (0.2% between most and least deprived areas) Achieving BP target value: gap narrowed to a small but significant residual difference pro less deprived but for DM a small inverse gap occured

Ashworth 2007a. The relationship between social deprivation and the quality of primary care: a national survey using the indicators from the UK quality and outcomes framework	Serial cross sectional Post contract 2004-2005 and 2005-2006	Total QOF score 147 indicators	Decreasing gap to a small but significant residual difference 2 years after introduction of QOF pro less deprived

Doran 2008a. Effect of financial incentives on inequalities in the delivery of primary clinical care in England: analysis of clinical activity indicators for the quality and outcomes framework	Serial cross sectional Post contract 2004-2005; 2005-2006 and 2006-2007	48 clinical activity indicators	Decreasing gap to a small but significant residual difference 3 years after introduction of QOF pro less deprived

***Ethnicity***			

Millet 2007b. Impact of a pay-for-performance incentive on support for smoking cessation and on smoking prevalence among people with diabetes	Longitudinal repeated measurements Pre- and post contract 2003 and 2005 Effects of ethnicity studied adjusting for age, sex, deprivation and practice level clustering.	Diabetes recorded smoking status smoking cessation advice smoking prevalence	Recorded smoking status: Increasing gap pro ethnic minorities Smoking cessation advice: Gap disappeared post contract Smoking: Decreasing gap remaining pro whites

Millet 2008c. Impact of pay for performance on ethnic disparities in intermediate outcomes for diabetes: longitudinal study	Longitudinal Repeated measurements Pre- and post contract 2000 and 2005-2006	Diabetes HbA1c measurement BP measurement	HbA1c& BP measurement: no gap documented post contract Achievement of BP levels Increasing gap pro Whites and South Asians Achievement HbA1c levels Increasing gap pro Whites

Millet 2007a Ethnic disparities in diabetes management and pay for performance in the UK: The Wandsworth prospective diabetes study	Longitudinal Repeated measurements Pre- and post contract 2003-2004 and 2005-2006 Effects of ethnicity studied adjusting for age, sex, deprivation and practice level clustering.	Diabetes/Hyperglycemia management & control Hyperlipidemia management & control Hypertension Management & Control	Achievement HbA1c target: Increasing gap pro whites Prescription OHA: Increasing gap pro ethnic minorities Prescription insulin Increasing gap pro whites Achievement cholesterol target Decreasing gap remaining pro ethnic minorities Prescription Lipid lowering drugs Gap Whites vs Black Caribeans disappeared Gap Whites vs Black Africans decreased remaining pro whites Gap Whites vs Bangladeshi occurred (pro Bangladeshi) Achievement BP target Increasing gap pro whites Prescription ACE inhibitors No gap documented post contract

Millet 2008b. Ethnic disparities in coronary heart disease management and pay for performance in the UK	Serial cross sectional Pre and post contract 2003 and 2005	CHD 10 indicators	Decreasing gap remaining pro whites Gap Asians vs Whites occurs pro Asians

Ashworth 2008. Effect of social deprivation on blood pressure monitoring and control in England: analysis of clinical activity indicators for the quality and outcomes framework	Serial cross sectional Post contract 2004-2005; 2005-2006 and 2006-2007	5 chronic conditions Blood pressure monitoring	Gap between least and most deprived areas narrowed to a negligible difference, with the proportion of ethnic minorities having the strongest confounding effect on BP monitoring

**Age**			

Millet 2008c. Impact of pay for performance on ethnic disparities in intermediate outcomes for diabetes: longitudinal study	Longitudinal Repeated measurements Pre- and post contract 2000 and 2005-2006	Diabetes HbA1c measured BP measured	Change in BP level: pro young Change in HbA1c level: pro old

Millet 2007b. Impact of a pay for performance incentive on support for smoking cessation and on smoking prevalence among people with diabetes.	Longitudinal Repeated measurements Pre- and post contract 2003 and 2005 Effects of age studied adjusting for sex, deprivation, ethnic background and practice level clustering.	Diabetes/recorded smoking status, smoking sessation advice, smoking prevalence	Recorded smoking status: gap disappears Smoking cessation advice: gap disappears Smoking: decreasing gap remaining pro pro old

McGovern 2008b. The effect of the UK incentive based contract on the management of patients with stroke in primary care	Serial cross sectional Pre and post contract 2000 and 2005	CHD 11 indicators	Decreasing gap from 9/11 to 7/11 CHD indicators

McGovern 2008. Introduction of a new incentive and target based contract for family physicians in the UK: good for older patients with diabetes but less good for women.	Serial cross sectional Pre and post contract 2000 and 2005	DM 8 indicators	Gap pro younger decreased (5/8 to 1/8 indicators pro young) Gap pro older increased (2/8 to 4/8 indicators pro old)

Simpson 2006. Effect of the UK incentive based contract on the management of patients with stroke in primary care.	Serial cross sectional Pre and post contract 2004 and 2005	CVD 9 indicators	Gap pro younger decreased (6/9 to 4/9 indicators pro young) Gap pro older increased (1/9 to 2/9 indicators pro old)

***Sex***			

Millet 2008c. Impact of pay for performance on ethnic disparities in intermediate outcomes for diabetes: longitudinal study	Longitudinal Repeated measurements Pre- and post contract 2000 and 2005-2006	Diabetes HbA1measured BP measured	Change in diastolic BP level: pro men Change in HbA1c level: pro women

Millet 2007b. Impact of a pay for performance incentive on support for smoking cessation and on smoking prevalence among people with diabetes.	Longitudinal Repeated measurements Pre- and post contract 2003 and 2005 Effects of sex studied adjusting for age, deprivation, ethnic background and practice level clustering.	Diabetes recorded smoking status smoking cessation advice smoking prevalence	Recorded smoking status: Decreasing gap Smoking sessation advice: No gap documented Smoking: Decreasing gap

McGovern 2008b. The effect of the UK incentive based contract on the management of patients with stroke in primary care	Serial cross sectional Pre and post contract 2000 and 2005	CHD 11 indicators	Increasing gap from 7/11 to 9/11 CHD indicators pro men

McGovern 2008. Introduction of a new incentive and target based contract for family physicians in the UK: good for older patients with diabetes but less good for women.	Serial cross sectional Pre and post contract 2000 and 2005	DM 8 indicators	Increasing gap from 2/8 to 5/8 DM indicators pro men

Simpson 2006. Effect of the UK incentive based contract on the management of patients with stroke in primary care.	Serial cross sectional Pre and post contract 2004 and 2005	CVD 9 indicators	Decreasing gap from 7/9 to 5/9 CVD indicators pro men

^1^CHD: Coronary Heart Disease

^2 ^DM: Diabetes Mellitus

^3^CVD: Cerebrovascular Disease

^4^BP: Blood Pressure

**Table 3:** Cross-sectional studies with one point of measurement after introduction of QOF

Study	Study design	Condition/Indicator	Cross sectional analysis after introduction of the Quality and Outcomes Framework
***Deprivation***			

Ashworth 2007b. Social deprivation and statin prescribing: a cross sectional analysis using data from the new UK general practitioner 'Quality and Outcomes framework'	Cross sectional Post contract 2004-2005	Prescription of statins (corrected for the prevalence of CVD and diabetes)	Pro deprived practices

Gulliford 2007 Achievement of metabolic targets for diabetes by English primary care practices under a new system of incentives	Cross sectional Post contract 2005	Hba1c achievement	Pro less deprived

Millet 2007c Diabetes prevalence, process of care and outcomes in relation to practice size, caseload and deprivation: national cross sectional study in primary care	Cross sectional Post contract Exact year not specified	18 diabetes indicators	Pro less deprived

Saxena 2007 practice size, caseload, deprivation and quality of care of patients with coronary heart disease, hypertension and stroke in primary care: national cross sectional study	Cross sectional Post contract 2004-2005	Prevalence CHD 26 CHD indicators	Equal prevalence Pro less deprived for process indicators requiring referral

Strong 2006 Socioeconomic deprivation, coronary heart disease prevalence and quality of care: a practice level analysis in Rotherham using data from the new UK general practitioner Quality and Outcomes Framework	Cross sectional Post contract 2004-2005	Prevalence CHD 11 CHD indicators	Higher prevalence in deprived areas 10/11 indicators = 1/11 pro less deprived

Sutton 2006 Determinants of primary medical care quality measured under the new UK contract: cross sectional study	Cross sectional Post contract 2004-2005	Total QOF score (corrected for practice characteristics)	Pro deprived

Mc Lean 2006 Deprivation and quality of primary care services: evidence for persistence of the inverse care law from the UK quality and outcomes framework.	Cross sectional Post contract 2005	22 QOF indicators	17/22 pro less deprived Simple process measures show less inequalities than complex process measures, intermediate outcome measures and measures of therapy

Doran 2006 Pay for performance programs in family practices in the United Kingdom	Cross sectional Post contract 2004-2005	Overall QOF achievement scores	Pro less deprived

Bottle Association between quality of primary care and hospitalization for coronary heart disease in England: national cross sectional study	Cross sectional Post contract 2004-2005	Hospital admission rates	Pro less deprived

Walters Ethnic density, physical illness, social deprivation and antidepressant prescribing in primary care: ecological study	Cross sectional Post contract 2004-2005	Prescription volumes of antidepressant drugs	Higher prescription volumes in deprived groups

Ashworth. The relationship between general practice characteristics and quality of care: a national survey of quality indicators used in the UK Quality and Outcomes Framework 2004-2005	Cross sectional Post contract 2004-2005	Overall QOF achievement scores	Pro less deprived

***Ethnicity***			

Ashworth 2007b Social deprivation and statin prescribing: a cross sectional analysis using data from the new UK general practitioner 'Quality and Outcomes framework'	Cross sectional Post contract 2004-2005	Prescription of statins (corrected for the prevalence of CVD and diabetes)	South Asians and Afro Caribeans < Whites despite their higher need for coronary healthcare

Gray 2007 Ethnicity and quality of diabetes care in a health system with universal coverage: population based cross sectional study in primary care	Cross sectional Post contract 2005-2006	13 diabetes indicators - 10 process measures - 3 outcome measures Controlled for age, sex and deprivation	process measures 6/10 = 3/10 blacks > whites 1/10 whites > blacks 2/10 SA > whites outcome measures 3/3 whites > blacks 2/3 whites > SA 1/3 SA > whites

Gulliford 2007 Achievement of metabolic targets for diabetes by English primary care practices under a new system of incentives	Cross sectional Post contract 2005	Hba1c	Pro whites Lower achievement rates in areas with a high proportion of ethnic minorities

Millet 2008 Ethnic disparities in blood pressure management in patients with hypertension after the introduction of pay for performance	Cross sectional Post contract 2005-2006	BP achievement levels Prevalence CVD	Whites > blacks SA > blacks SA > blacks SA > whites Blacks > SA Whites > SA Blacks > whites SA > whites

Doran 2006 Pay for performance programs in family practices in the United Kingdom	Cross sectional Post contract 2004-2005	Overall QOF achievement scores	No significant differences

Walters Ethnic density, physical illness, social deprivation and antidepressant prescribing in primary care: ecological study	Cross sectional Post contract 2004-2005	Prescription volumes of antidepressant drugs	Lower prescription volumes in populations with high Black or South Asian ethnicity

**Age**			

Ashworth 2007b Social deprivation and statin prescribing: a cross sectional analysis using data from the new UK general practitioner 'Quality and Outcomes framework'	Cross sectional Post contract 2004-2005	Prescription of statins (corrected for the prevalence of CVD and diabetes)	Pro young (< 75)

Doran 2006 Pay for performance programs in family practices in the United Kingdom	Cross sectional Post contract 2004-2005	Overall QOF achievement scores	Pro young (< 65)

***Sex***			

Ashworth 2007b Social deprivation and statin prescribing: a cross sectional analysis using data from the new UK general practitioner 'Quality and Outcomes framework'	Cross sectional Post contract 2004-2005	Prescription of statins (corrected for the prevalence of CVD and diabetes)	7/10 pro male

Doran 2006 Pay for performance programs in family practices in the United Kingdom	Cross sectional Post contract 2004-2005	Overall QOF achievement scores	=

### Data source, level of analysis and geographical area

Sixteen of the 27 studies used the QOF database, which contains uniform quality achievement data from almost all general practices in the UK [10,35-37,39-48,51,52]. QOF data are registered at the practice level. Ten studies used databases related to QOF that provide quality achievement data at the level of the individual such as the Wandsworth primary care based register [28-30,32,42,49] or the SPICE (Scottish Programme for improving clinical effectiveness in primary care) database [33,34,38,53]. One study also used data from the Health Survey for England based on patient interviews. Of the 27 included studies, twelve [28-31,33,34,38,42,49,50,53] analysed data at the patient level, 14 [10,35-37,39-41,43-47,51,52] analysed data at the practice level and one study analysed data at the level of the primary care trust [48]. Nineteen of the 27 studies were conducted on data from England [10,28-32,35-37,39-41,44,47-52], of which 6 publications were based on data from the geographical area of Wandsworth (London) [28-30,32,49,50]. Six studies were conducted on Scottish data [33,34,38,45,46,53] and two studies combined data from Scotland and England [42,43].

### Patient groups

Differences between socio-economic groups of patients received the most attention. Although all publications refer to socio-economic differences in some way (for example as a covariate in a multivariate analysis), only 19 studies [29,31,33-47,51]) report results on the impact of socio-economic differences or include enough detailed information in their tables to derive conclusions on the influence of socio-economic status on the reported indicators. Socio-economic status is estimated using the Index of Multiple Deprivation (IMD) (15 studies) [29,30,33-47,51], the DEPCAT score (3 studies) [33,34,38] or a derived variable for occupational class [31]. The IMD and DEPCAT scores are based on a broad range of indicators that determine the level of deprivation of a geographical area. They are used as a proxy for the social status of the patient because information on the individual level, e.g., educational level, is often not available or unreliable. Seven of the 16 studies [29,30,33,34,38,42,43] use the deprivation score of the area where the patient lives as a proxy for the patient's social status, and the other studies assign to the patient the deprivation score of the area where the practice is located, which is not always the area where the patient lives [36,37,40,41,44-47]. Occupational class was derived at the patient level and was defined into two groups: non-manual and manual occupations [31].

A smaller number of studies (11/27) address differences between ethnic groups: 6/11 use the patient's self-rated ethnic origin [28-30,32,49,50] and 5/11 use the ethnic composition of the area were the practice is located [10,35-37,39,41,47].

Finally, 7/27 studies [29,30,33,34,38,40,47] look at gender differences and differences between age groups.

### The conceptualisation of equity

In 14 of the 27 studies, the evaluation of equity is an explicit aim (or one of the aims) of the study [29,31-36,38,39,42,46,50-52]. The other studies report results related to equity as additional information or include tables with enough detail to allow the deduction of equity related results.

To evaluate equity in access to care, information on the profile of both users and non-users is essential. None of the selected studies contains information concerning non-users.

Most of the selected studies focus on differences in treatment (15/27) [28-35,39,40,42,44,49,51,52] such as statin prescribing and/or (intermediate) treatment outcomes (19/27) [28-33,35,36,41-44,47,49-53] such as achievement levels for HbA1c or blood pressure control. Four of the 27 studies use total QOF scores as an outcome parameter [37,38,45,51]. In 3 other studies, inequity in exception reporting is investigated [10,52,53]. None of the selected studies take the concept of "need" (defined as normative need, felt need or expressed need) into consideration. In the majority of the studies, the authors implicitly adopt a comparative approach to need: when variations are found between the treatment rates and outcomes of two groups of patients with the same condition, inequity is presumed without questioning the appropriateness of the quality indicators for the specific groups [6].

### Evolution of equity of access to care

None of the publications assessed dimensions of access to health care. Neither the availability of equal services nor equal quality of services for people in equal need is addressed in the selected papers, nor is the aspect of variations in personal inconvenience, cost or availability of information for patients from different backgrounds.

### Evolution of equity in treatment and (intermediate) outcomes (see Tables 2 and 3)

An age gap in the quality of health care for coronary heart disease (CHD), diabetes and cerebrovascular disease (CVD) was documented before the implementation of QOF [30,33,34,38]. For the majority of the quality indicators (21/33 indicators), an inequity in favour of younger patients was detected. QOF succeeded in reducing this age gap by improving the quality of health care for the oldest patients more than for the younger patients [33,34,38]. For 5 of the 33 indicators, a bias in favour of older patients was detected before the introduction of QOF. Concerning the recording of smoking status and giving smoking cessation advice, there seems to be no effect of age on their positive evolution since the introduction of QOF. For the 3 other indicators, the older patient bias persisted [34,38]. For 2 additional indicators, a new older patient bias occurred.

Two cross-sectional studies with one measurement after the introduction of QOF described an age gap favouring the young for prescription of statins [37] and for overall QOF achievement scores [47].

Men seem to have benefited more from QOF than women. Before the introduction of the contract, men scored significantly better on the quality of care for CHD (7/11 indicators), CVD (7/9 indicators) and diabetes (2/8 indicators) [33,34,38]. After QOF introduction, all the CHD and diabetes indicators with a difference favouring men persisted, and additionally, a pro-male inequity occurred for a number of other indicators (2/11 for CHD and 3/8 for diabetes) [33,34]. For CVD, the gap became smaller but remained in favour of men [38].

In diabetics, Millet et al. observed a difference favouring women for the recording of smoking status and smoking prevalence but not for giving smoking cessation advice. QOF introduction resulted in a larger increase in the quality of care for men than for women, decreasing this gap [30]. A cross-sectional analysis with one measurement after introduction of the contract described equal overall achievement rates for men and women [47].

Pre-QOF, a difference between deprived and less deprived areas was found for a relatively small number of quality indicators related to CHD, diabetes and CVD (e.g., 1/11 indicators for CHD). Some indicators are even in favour of the patients in the most deprived areas (e.g., HbA1c recorded) [31,33,34,38]. Shortly after the introduction of QOF, some studies described a slight increase in inequity e.g., additional inequity for 3/9 CVD indicators and 1/4 CHD indicators [31,38]. Also, greater variation in achievement between practices was found with greater deprivation [35]. However, the gap existing in the first year after the introduction of QOF narrowed in the years after to almost negligible differences for all the described conditions [37,35]. For blood pressure control in diabetic patients, a small inverse gap occurred [36]. This gain can be attributed to greater quality improvements in practices in more deprived areas (made possible because of their poorer initial performance, rather than their location in a deprived area). Nevertheless, for some individual indicators (e.g., patients with epilepsy who were seizure-free for > 12 months), large differences remain (for 5 of the 147 measured QOF quality indicators, the difference between the least and most deprived areas is larger than 10% [37]), and the poorest performing practices remain concentrated in the most deprived areas [35]).

Cross-sectional data after the introduction of QOF indicate that overall quality as measured by QOF remains in favour of the affluent [47,51]. However, Sutton et al. [45] report an inverse gap for overall QOF scores when they corrected for practice characteristics such as team size and composition, financial incentives, accreditation, training status and average age of GP. At the level of individual indicators or diseases, negligible or only small differences in favour of the less deprived are observed [41-46,48]. One study found higher statin prescription rates in practices serving more deprived populations even after adjusting for the increased prevalence of cardiovascular disease and diabetes [40]. Another study described higher prescription volumes of antidepressants in practices serving more deprived populations [39]

Concerning ethnicity, it seems that the impact of QOF implementation is different for different ethnic groups. Both before and after the implementation of QOF, the results regarding ethnic differences are scattered. Studies have focused mainly on CDH and diabetes. Pre-QOF, CDH patients of South Asian origin had better-controlled cholesterol than white or black patients. After the introduction of QOF, they even scored better in 3 additional aspects of care. The small gap between black and white people narrowed even further after the implementation of QOF (from 2 to 1 out of 10 indicators; BP control and statin prescribing, of which the latter remained) [32]. For some aspects of diabetes care, the existing gap between whites and blacks/Indians (recording of smoking status), and between whites and Indians (achieving HbA1c target) increased, favouring non-whites. For blood pressure and HbA1c level achievement, the gap seems to favour whites compared to blacks [29]. In some cases an inverse equity gap occurs: e.g., from a pro-white to a pro-Pakistani inequity in recorded smoking status [30]. Five studies report on cross-sectional data after the introduction of the contract. On the level of individual indicators, results are scattered [39-41,47,49,50]. However, overall achievement rates show no relationship with ethnicity [47].

### (In)equity in exception reporting

Three studies [10,38,52] look at the possible impact of exception reporting on inequity in health care. One study reports that diabetics living in deprived areas are more likely to be "exception reported" [52]. A study on outcomes in CVD found no significant association between sex, age, deprivation and the level of exception reporting [38]. The most recent and most comprehensive study on this topic [10] reports that the characteristics of patients (e.g., gender and socioeconomic position) explain only 2.7% of the variance in exception reporting. The authors conclude that "Exception reporting brings substantial benefits to pay-for-performance programs, providing that the process has been used appropriately" and that "Rates of exception reporting have generally been low, with little evidence of widespread gaming."[10]. However, it can be argued that although the exclusion system succeeds in not being socially selective, it does not succeed in rewarding the additional work required in deprived areas and in that way it might still contribute to the inverse care law [46].

## Discussion

There is widespread concern that the focus on quality improvement systems driven by financial incentives may lead to a widening of the existing inequity in health care. In this paper, we aimed to describe the evolution of pre-existing (in)equity in health care in the period after the introduction of the Quality and Outcomes Framework (QOF) in the UK and to describe any (in)equities in exception reporting. A systematic literature review was set up, and the selected publications were analysed using a conceptual framework regarding equity. This framework developed by the researchers is based on the existing equity literature and builds on the distinction between equity in access to care, equity in treatment and equity in treatment outcomes.

A systematic search of MEDLINE and Econlit resulted in 317 abstracts. They were screened for their focus on the Quality and Outcomes Framework and for explicit references to equity related concepts or to patient subgroups. Finally, twenty-seven publications were selected for analysis.

Equal access to care for all patients is an essential prerequisite to equal health care. However, none of the selected publications compares the profile of users versus non-users of care, making it impossible to assess the impact of QOF on access to care. This is probably influenced by the context of the UK's health system where access to primary care services is almost universal because only a very small minority of patients is not registered. Despite the universality of the system, some specific population groups still find it difficult to register with a GP. For instance, homeless people often do not know that they have to register or are scared off by the complexity of the registration procedure [54]. Furthermore, being registered does not necessarily mean that patients do not experience problems in accessing care. Several studies identified barriers in the accessibility of the health care system that go far beyond being registered or having to pay or not. Also, characteristics of care related to the design and delivery of health care and the features and skills of the providers may or may not encourage or enable patients to use medical care services. Financially-driven quality improvement systems using purely biomedical indicators may lead to the loss of important aspects of health care quality such as trust and high-quality empathic communication [55,56]. It has been suggested that QOF might have changed the nature of the practitioner-patient consultation with, for instance, a decline in personal/relational continuity of care between doctors and patients [57]. To assess equity in access to care, information on the number and type of users and non-users (registered or not) is indispensable, and there is a need for studies researching this aspect of equity.

Most of the selected studies provide information on equity in treatment and in treatment outcomes. With the introduction of QOF, the quality of care in the UK generally improved (at least for the conditions included in QOF), and for the majority of the observed indicators, all citizens have benefited from this improvement. However, the extent to which different patient groups benefit tends to vary and to be highly dependent on the type and complexity of the indicator(s) under study, the observed patient group, the characteristics of the study (such as design, level of analysis, covariates) and the level of detail of the studied indicators.

We can state that the introduction of QOF has benefited the aged and males. Regarding ethnicity and deprivation, it is almost impossible to draw general conclusions. At the level of total QOF score, ethnicity appeared to be of no influence [47]. For deprivation, small but significant residual differences were observed after the introduction of QOF favouring less deprived groups [37]. However, after correcting for practice characteristics, the influence of deprivation was no longer observed, indicating that the small but existing differences between socio-economic groups are mainly due to differences at the practice level. Practices in affluent areas are possibly better trained and better surrounded [45].

According to the inverse equity hypothesis formulated in 2000 by Victora et al., affluent groups in society preferentially benefit from new interventions, leading to an initial increase in inequality. Deprived groups only begin to benefit once affluent groups have extracted maximum benefit. Health inequalities ultimately diminish because deprived groups start with a lower baseline level of health and health care uptake and have higher potential gains [10,35,58]. The above results cannot unanimously confirm the first part of the hypothesis, but neither can they refute it as none of the selected studies reports on more than 2 years after QOF implementation.

Equity builds on the concept of need: equal health care means equal access, treatment and treatment outcomes for people in equal need. Considering the concept of "need", the authors of the selected papers (implicitly) adopt a comparative approach. Characteristic of this approach is that it makes no judgments about the appropriateness of the targets for the two groups. However, the absence of differences in the level of achievement between social, gender or age groups cannot automatically be interpreted as an absence of inequities because the need for care might be greater in some patient groups because of higher individual complexity [6]. Failure to align the delivery of health care to the needs of the community may result in the classic mismatch described by Tudor Hart in which those most in need of health care receive the least and the poorest quality services.

Not only is there need for differentiation in indicator achievement goals depending on the needs of the patient group, questions may also be asked about the relevance and the completeness of the QOF indicators. QOF indicators mainly focus on process indicators and intermediate outcomes. However, the extent to which equity in intermediate outcomes or process indicators predicts final outcomes remains unknown, as does the extent to which inequities in health care predict inequities in health. Furthermore, it has been documented that a comprehensive approach to quality is needed, including medical, contextual and policy evidence [59], especially in primary care where providers are confronted with the complexity of the individual and not only with the complexity of a single disease. In cases of multimorbidity, one may question the relevance of disease-specific targets as proposed in the Quality and Outcomes framework [60,61]. Also, at the level of the relationship between doctor and patient, the QOF indicators do not capture the complexity of this interaction. For example, the finding that higher volumes of statin and antidepressants are prescribed in deprived areas might also indicate that in deprived areas, a pill-oriented-strategy is preferred by providers (and counted by QOF), rather than a behaviour-oriented strategy. Or because building a trustful relationship with high-quality communication is more complex for deprived patients and patients with a different ethnic background, this might result in an increase of the health care inequity gap. Testing this type of hypothesis in prospective studies at the patient level is essential to assessing the impact of QOF on the equity of health care. In the continuous development and revision of the QOF indicators by the NHS and its partners, equity has to be a point of particular interest, and the impact of the chosen indicators on equitable care has to be monitored carefully.

Several methodological limitations complicate the formulation of the evidence, prompting utmost prudence in interpreting and generalising the results. Firstly, the papers in the study were retrieved from Medline and Econlit. It is possible that other databases could have identified additional publications. However, a search in additional databases (Embase, Web of Science, Cochrane, Psychinfo) on 17/01/2011 using the same timeframe did not reveal any new papers. Also, a screen of unpublished documents and of grey literature could have added new information. Secondly, because of the large variety in the variables under study, it was impossible to perform any statistical meta-analysis on the reported data. Therefore, this paper is restricted to a systematic description of the equity related information from the selected publications.

Thirdly, the selected publications use databases at the practice level and not at the patient level, which makes it impossible to describe the health care utilisation, treatment tracks and outcomes of individual patients. Moreover, the reported studies often use the practice as the level of analysis and/or use area level scores of deprivation as a proxy for the socioeconomic status of the patient, assuming that the eventual associations observed at the practice or area level reflect the same association at the individual level. This may not be true, a problem known as "ecological fallacy" [43,44,52]

In relation to indispensable developments towards a more comprehensive primary care system such as the development of the medical home model [62] in the US, it seems to be important to not only monitor explicitly social indicators in order to assess effects on equity. Moreover QOF type drivers may influence the nature of the doctor patient interaction shifting the focus to disease oriented care especially when mainly disease oriented economic incentives are included in the care process, hereby possibly counteracting patient centred and comprehensive care.

This study makes it clear that any change in a health care system should be analysed taking into account the historical, sociological, economic and cultural context. Replicating QOF in another country with different health care and payment systems could have a completely different outcome.

## Conclusions

Although this study is placed within the context of the QOF, its implications for health policy, quality of care provision and equity issues are applicable to many health care systems. A first point of attention when evaluating health care reforms is the extent to which different population groups find their way to the health care system. Not only is the coverage rate important, but so is more detailed knowledge on the differential use of services and the barriers patients experience in accessing the care they need. Attention to non-financial barriers such as transparency of the system, caregiver characteristics and waiting lists is therefore of utmost importance. Qualitative studies introducing the vision and experiences of care providers and patients might contribute to an explanation of the mechanisms that lead to the observed differences. Secondly, differential targets for specific patient groups should be considered. Without them, equal achievement levels could give the impression of equity in care when there might actually be inequity (e.g., equal blood pressure measurement rates in different ethnic groups might indicate an inequity in health care because of unequal prevalence of hypertension between the groups). Thirdly, it is important to look for indicators that embrace complexity. Non-disease specific, patient-centred indicators such as functional status and quality of life might be useful in this context. Fourthly, it is of utmost importance to collect information at the patient level and to create possibilities for monitoring individual health care utilisation tracks. Finally, we think it is important to evaluate P4Q initiatives in a broader health systems impact assessment strategy in which equity is a criterion equal in importance to other criteria such as cost-effectiveness [63]. The conceptual framework provided herein is a guide to developing new evidence and utilising existing evidence for evaluating the equity dimension of healthcare systems.

## Competing interests

The authors declare that they have no competing interests.

## Authors' contributions

PB and SW conceptualized the framework to address equity, selected the eventual publications, extracted and analyzed the data and drafted the manuscript. DDS assisted in the systematic search of 317 publications and the exclusion of the first 215 publications. JDM and LA participated in the design of the study and provided continuous supervision and feedback. All authors read and approved the final manuscript.

## Pre-publication history

The pre-publication history for this paper can be accessed here:

http://www.biomedcentral.com/1472-6963/11/209/prepub
